# Prognostic impact of final kissing balloon technique after crossover stenting for the left main coronary artery: from the AOI-LMCA registry

**DOI:** 10.1007/s12928-018-0522-0

**Published:** 2018-04-24

**Authors:** Koji Nishida, Mamoru Toyofuku, Takeshi Morimoto, Masanobu Ohya, Yasushi Fuku, Hirooki Higami, Kyohei Yamaji, Hiromi Muranishi, Yuhei Yamaji, Daisuke Furukawa, Tomohisa Tada, Euihong Ko, Kazushige Kadota, Kenji Ando, Hiroki Sakamoto, Takashi Tamura, Kazuya Kawai, Takeshi Kimura, The AOI LMCA Stenting Registry Investigators

**Affiliations:** 1 Department of Cardiology, Chikamori Hospital, Kochi, Japan; 2 Department of Cardiology, Japanese Red Cross Society Wakayama Medical Center, Wakayama, Japan; 3 Department of Clinical Epidemiology, Hyogo College of Medicine, Nishinomiya, Japan; 4 Department of Cardiology, Kurashiki Central Hospital, Kurashiki, Japan; 5 Department of Cardiovascular Medicine, Graduate School of Medicine, Kyoto University, 54 Shogoin Kawahara-cho, Sakyo-ku, Kyoto, 606-8507 Japan; 6 Department of Cardiology, Kokura Memorial Hospital, Kokura, Japan; 7 Department of Cardiology, Shizuoka General Hospital, Shizuoka, Japan

**Keywords:** Left main coronary artery, Kissing balloon technique, One-stent strategy

## Abstract

It is still uncertain whether the final kissing balloon technique (FKBT) is mandatory after crossover stenting for the left main coronary artery (LMCA). Assessing Optimal Percutaneous Coronary Intervention for LMCA (AOI-LMCA) registry, a 6-center retrospective registry, enrolled 1809 consecutive patients for LMCA stenting in Japan. In the present analysis, 5-year clinical outcomes were compared between non-FKBT (*n* = 160) and FKBT (*n* = 578) groups in patients treated with crossover stenting with drug-eluting stents from the LMCA to the left anterior descending artery. Propensity score-matched analysis was also performed in 160 patient pairs. In the entire study population as well as in the propensity-matched population, the cumulative 5-year incidence of the primary outcome measure (target lesion revascularization: TLR) was not significantly different between the FKBT and non-FKBT groups (10.7 versus 14.3%, *P* = 0.49, and 11.8 versus 14.3%, *P* = 0.53, respectively). In the sensitivity analysis by the multivariable Cox proportional hazard model, the effect of FKBT relative to non-FKBT for TLR remained insignificant (adjusted HR 0.89, 95% CI 0.47–1.69, *P* = 0.72). Regarding the TLR location, there were no significant differences in the cumulative incidences of TLR for LMCA-only, for the main branch, and for the side branch between the 2 groups (2.2 versus 1.3%, *P* = 0.93, 11.8 versus 9.1%, *P* = 0.71, and 8.2 versus 7.6%, *P* = 0.82, respectively). FKBT after a 1-stent strategy for LMCA crossover stenting did not affect TLR and other clinical outcomes during 5-year follow-up.

**Clinical Trial Registration:** Assessing Optimal Percutaneous Coronary Intervention for Left Main Coronary Artery Stenting Registry (AOI LMCA Stenting Registry). http://www.umin.ac.jp/ctr/index/htm/. Unique Identifier: UMIN000014706.

**Electronic supplementary material:**

The online version of this article (10.1007/s12928-018-0522-0) contains supplementary material, which is available to authorized users.

## Introduction

The current international guidelines have recommended coronary artery bypass grafting (CABG) as a class 1 indication in patients with left main coronary artery (LMCA) disease [1–3]. Percutaneous coronary intervention (PCI) has been more and more frequently performed in patients with LMCA disease with a low-moderate SYNTAX (SYNergy between percutaneous coronary intervention with TAXus and cardiac surgery) score as an alternative to CABG, due to improvements of drug-eluting stents (DES) and advancing technique [4, 5]. A 1-stent strategy is currently considered a standard stenting strategy for LMCA bifurcation lesions, because a 2-stent strategy is associated with higher rates of adverse events such as target lesion revascularization (TLR), and stent thrombosis (ST) [6, 7]. However, the role of final kissing balloon technique (FKBT) after crossover stenting for the main branch is controversial for lesions at any bifurcation [8]. Particularly, the effects of FKBT for LMCA disease have not been adequately assessed in previous reports [9–13]. Therefore, we sought to compare the long-term clinical outcomes between the 2 groups of patients with and without FKBT after crossover DES stenting from LMCA to the left anterior descending artery (LAD), using data from a large multicenter registry in Japan.

## Methods

### Study design and patient population

The AOI-LMCA (Assessing Optimal percutaneous coronary Intervention for Left Main Coronary Artery) stenting registry is a retrospective, multicenter registry that enrolled 1809 consecutive patients who underwent LMCA stenting with bare-metal stents or DES in 6 Japanese hospitals experienced with LMCA stenting between November 2004 and December 2012. The protocol and details of patient enrollment have been described elsewhere [6].

**Fig. 1:**
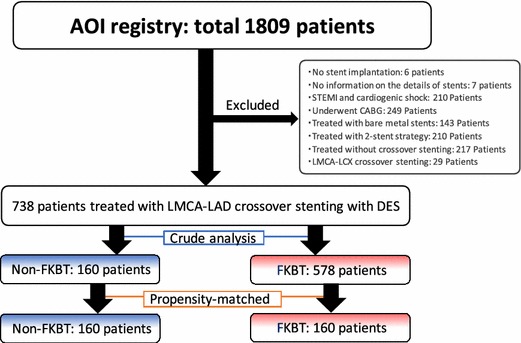
Study flow chart. *AOI* assessing optimal percutaneous coronary intervention for left main coronary artery stenting, *CABG* coronary artery bypass grafting, *DES* drug-eluting stents, *FKBT* final kissing balloon technique, *LAD* left anterior descending coronary artery, *LCX* left circumflex coronary artery, *LMCA* left main coronary artery, *STEMI* ST-segment elevation myocardial infarction

The current study population comprised 738 patients treated with crossover DES stenting as a 1-stent strategy from LMCA to LAD. Five-year clinical outcomes were compared between the non-FKBT group (*N* = 160) and the FKBT group (*N* = 578) after excluding those patients who had ST-elevation myocardial infarction (MI) with cardiogenic shock, or LMCA–left circumflex coronary artery (LCX) crossover stenting (Fig. 1).

Stenting strategies and use of FKBT were left to the discretion of the operators in 5 of 6 participating centers, except for one center in which culotte was the default bifurcation stenting strategy and FKBT after crossover stenting was mandatory. The Medina classification was used to assess each bifurcation lesion, and true bifurcation was defined as Medina classification (1, 1, 1), (1, 0, 1), or (0, 1, 1) [14]. TLR-main branch was defined as TLR from the LMCA to the LAD, and TLR-side branch was defined as TLR involving the LCX ostium only. After the procedure, aspirin (100 mg/day) and ticlopidine (200 mg/day) or clopidogrel (75 mg/day) were to be prescribed in all patients. The recommended duration of dual antiplatelet therapy (DAPT) was at least 8–12 months after DES implantation. Actual DAPT duration in each patient was determined by each cardiologist based on the institutional protocol. Follow-up data collection was performed by a review of medical records and follow-up contact with patients and/or their relatives. The study protocol was approved by the institutional review board in each participating center. Written informed consent from each patient was waived in this retrospective study, because we used clinical information obtained in the routine clinical practice and no patients refused to participate in the study when contacted for follow-up.

### Endpoints

The primary outcome measure in the present analysis was TLR at 5-year. Secondary outcome measures included all-cause death, cardiac death, sudden death, MI, definite or probable ST, stroke, any coronary revascularization, or major adverse cardiovascular events (MACE: cardiac death, MI, or TLR) at 5-year. Definitions for each endpoint have been previously described in detail [6].

### Statistical analysis

Categorical variables are presented as counts and percentages, and compared using Chi-squared or Fisher’s exact tests. Continuous variables are expressed as mean ± standard deviation or medians with interquartile range. Continuous variables were compared using Student’s *t* test or the Wilcoxon rank-sum test based on distributions. The cumulative incidence of each endpoint was estimated using the Kaplan–Meier method, and the curves of the 2 groups were compared by the log-rank test.

To adjust the potential confounding for the choice of FKBT, we evaluated the effects of FKBT strategy relative to non-FKBT strategy in a propensity score-matched population. A logistic regression model was used to develop propensity scores for the choice of FKBT strategy with 15 independent variables relevant to the decision regarding FKBT strategy listed in Tables 1 and 2. The propensity score was then calculated by summing up all coefficient multiples for the corresponding variables (Supplemental Table). To create the propensity score-matched cohort, patients without FKBT were matched to those with FKBT using a 1:1 greedy matching technique [15]. The cumulative incidences of clinical events were compared between FKBT and non-FKBT strategies in the propensity score-matched cohort.

**Table 1:** Baseline patient characteristics in the entire study population and in the propensity-matched population: FKBT versus non-FKBT

	Entire study population			Propensity-matched population		
	FKBT (*n* = 578)	Non-FKBT (*n* = 160)	*P* value	FKBT (*n* = 160)	Non-FKBT (*n* = 160)	*P* value
Age (years)	72 ± 10	73 ± 11	0.34	74 ± 9	73 ± 11	0.52
Age ≥ 80 years^a,b^	141 (24%)	48 (30%)	0.15	44 (28%)	48 (30%)	0.62
Male	441 (76%)	117 (73%)	0.41	120 (75%)	117 (73%)	0.7
Hypertension	436 (75%)	131 (82%)	0.09	115 (72%)	131 (82%)	0.03
Diabetes mellitus^a,b^	258 (45%)	78 (49%)	0.36	67 (42%)	78 (49%)	0.22
Insulin-treated diabetes	62 (11%)	23 (14%)	0.2	14 (9%)	23 (14%)	0.12
Dyslipidemia	328 (57%)	106 (66%)	0.03	91 (57%)	106 (66%)	0.09
Current smoker	80 (14%)	32 (20%)	0.06	28 (18%)	32 (20%)	0.57
eGFR (mL/min/1.73 m^2^)	60.8 ± 23.1	58.6 ± 24.3	0.31	58.7 ± 21.4	58.6 ± 24.3	0.96
Hemodialysis^a,b^	26 (4.5%)	14 (8.8%)	0.036	11 (6.9%)	14 (8.8%)	0.53
eGFR < 60 mL/min/1.73 m^2^ and non-hemodialysis^a,b^	229 (40%)	68 (43%)	0.51	66 (41%)	68 (43%)	0.82
Previous PCI^a^	286 (50%)	75 (47%)	0.56	76 (48%)	75 (47%)	0.91
Previous myocardial infarction	183 (32%)	46 (29%)	0.48	42 (26%)	46 (29%)	0.62
Previous heart failure^a,b^	72 (13%)	18 (11%)	0.68	22 (14%)	18 (11%)	0.5
Malignancy^a^	58 (10%)	19 (12%)	0.5	12 (7.5%)	19 (12%)	0.19
Stroke^a^	80 (14%)	22 (14%)	0.98	23 (14%)	22 (14%)	0.87
Peripheral vascular disease^a^	64 (11%)	36 (23%)	<0.0001	21 (13%)	36 (23%)	0.03
Euro score	4.2 ± 2.4	3.9 ± 2.5	0.34	5.1 ± 3.0	5.5 ± 3.5	0.34
Clinical presentation			0.7			0.57
Stable angina pectoris	469 (81%)	132 (83%)		128 (80%)	132 (83%)	
UAP/NSTEMI^a,b^	109 (19%)	28 (18%)		32 (20%)	28 (18%)	
Decompensated heart failure	38 (6.6%)	12 (7.6%)	0.66	8 (5.0%)	12 (7.6%)	0.34
Medication						
Aspirin	565 (98%)	155 (98%)	0.74	157 (98%)	155 (98%)	0.7
Thienopyridine	559 (97%)	155 (98%)	0.77	157 (98%)	155 (98%)	0.7
Warfarin	42 (7.3%)	11 (6.9%)	0.87	16 (10.0%)	11 (6.9%)	0.32
Statins^a^	410 (71%)	107 (67%)	0.32	111 (69%)	107 (67%)	0.63
β-Blockers^a^	161 (28%)	48 (30%)	0.59	43 (27%)	48 (30%)	0.54
ACE-I/ARB^a^	337 (58%)	95 (59%)	0.81	88 (55%)	95 (59%)	0.43
Proton pump inhibitors	272 (47%)	61 (38%)	0.047	69 (43%)	61 (38%)	0.39
H_2_-blocker	83 (14%)	23 (15%)	0.99	22 (14%)	23 (15%)	0.85
Time period^a,b^			0.04			0.01
Wave 1: 2004–2006 (bare-metal stent period)	88 (15%)	37 (23%)		37 (23%)	27 (17%)	
Wave 2: 2007–2009 (G1-DES period)	213 (37%)	47 (29%)		44 (28%)	69 (43%)	
Wave 3: 2010–2012 (G2-DES period)	277 (48%)	76 (48%)		79 (49%)	64 (40%)	
Institute^a,b^			<0.0001			0.9
1	39 (6.7%)	23 (14%)		22 (14%)	23 (14%)	
2	63 (11%)	53 (33%)		50 (31%)	53 (33%)	
3	349 (60%)	22 (14%)		22 (14%)	22 (14%)	
4	94 (16%)	50 (31%)		56 (35%)	50 (31%)	
5	25 (4.3%)	8 (5.0%)		5 (3.1%)	8 (5.0%)	
6	8 (1.4%)	4 (2.5%)		5 (3.1%)	4 (2.5%)	

*eGFR* estimated glomerular filtration rate, *PCI* percutaneous coronary intervention, *UAP* unstable angina pectoris, *NSTEMI* non-ST-segment elevation myocardial infarction, *ACE-I* angiotensin-converting enzyme inhibitor, *ARB* angiotensin II receptor blocker, *G1-DES* first-generation drug-eluting stent, *G2-DES* second-generation drug-eluting stent

^a^Potential independent risk-adjusting variables selected for Cox proportional hazards models

^b^Daggers indicate the variables selected for propensity score matching

**Table 2:** Baseline lesion and procedural characteristics in the entire study population and in the propensity-matched population: non-FKBT versus FKBT

	Entire study population			Propensity-matched population		
	FKBT (*n* = 578)	Non-FKBT (*n* = 160)	*P* value	FKBT (*n* = 160)	Non-FKBT (*n* = 160)	*P* value
Lesion characteristic						
CTO in the RCA^a,b^	75 (13%)	20 (13%)	0.87	18 (11%)	20 (13%)	0.73
SYNTAX score	26.0 ± 9.4	27.2 ± 9.7	0.13	25.6 ± 8.8	27.3 ± 9.7	0.1
Bifurcation lesion	539 (93%)	146 (91%)	0.39	142 (89%)	146 (91%)	0.46
True bifurcation^a,b^	206 (36%)	59 (37%)	0.77	62 (39%)	59 (37%)	0.73
Extent of coronary artery disease			0.13			
Left main only	43 (7%)	13 (8%)		15 (9.4%)	13 (8.1%)	
Left main + 1 vessel	222 (38%)	47 (29%)		54 (34%)	47 (29%)	
Left main + 2 vessels	212 (37%)	62 (39%)		65 (41%)	62 (39%)	
Left main + 3 vessels	101 (18%)	38 (24%)		26 (16%)	38 (24%)	
Multi-vessel (left main + ≥ 2 vessels)^a,b^	313 (54%)	100 (63%)	0.06	91 (57%)	100 (63%)	0.31
Medina classification			0.03			0.28
(1, 0, 0)	76 (13%)	19 (12%)		23 (14%)	19 (12%)	
(0, 1, 0)	31 (5.4%)	7 (4.4%)		7 (4.4%)	7 (4.4%)	
(0, 0, 1)	3 (0.5%)	6 (3.8%)		0 (0%)	6 (3.8%)	
(1, 1, 0)	221 (38%)	52 (33%)		50 (31%)	55 (34%)	
(1, 0, 1)	21 (3.6%)	3 (1.9%)		6 (3.8%)	3 (1.9%)	
(0, 1, 1)	5 (0.9%)	4 (2.5%)		3 (1.9%)	5 (3.1%)	
(1, 1, 1)	169 (29%)	50 (31%)		53 (33%)	51 (32%)	
True trifurcation	71 (13%)	27 (17%)	0.15	23 (15%)	27 (17%)	0.57
Calcified lesion^a,b^	91 (16%)	18 (11%)	0.16	18 (11%)	18 (11%)	1.0
In-stent restenosis lesion	15 (2.6%)	4 (2.5%)	0.94	4 (2.5%)	4 (2.5%)	0.99
Procedural characteristic						
Arterial access site			0.04			0.90
Femoral	393 (68%)	122 (76%)		123 (77%)	122 (76%)	
Radial or brachial	185 (32%)	38 (24%)		37 (23%)	38 (24%)	
Use of mechanical support						
IABP	31 (5.4%)	10 (6.2%)	0.67	16 (10.0%)	10 (6.3%)	0.22
PCPS	2 (0.3%)	1 (0.6%)	0.63	1 (0.6%)	1 (0.6%)	1.0
Use of rotablator	42 (7.3%)	17 (11%)	0.17	11 (6.9%)	17 (11%)	0.24
Stent types			0.23			0.13
G1-DES	342 (59%)	103 (64%)		90 (56%)	103 (64%)	
G2-DES^a,b^	236 (41%)	57 (36%)		70 (44%)	57 (36%)	
Use of intracoronary imaging modalities						
IVUS^a,b^	409 (71%)	134 (84%)	0.001	127 (79%)	134 (84%)	0.31
OCT	24 (4.2%)	6 (3.8%)	0.82	15 (9.4%)	6 (3.8%)	0.04
None	145 (25%)	20 (13%)	0.001	18 (11%)	20 (13%)	0.73
Proximal optimization technique	119 (21%)	13 (8.1%)	0.001	26 (16%)	13 (8.1%)	0.03
Number of stents per lesion	1.3 ± 0.5	1.5 ± 0.7	0.001	1.3 ± 0.6	1.5 ± 0.7	0.07
Stent size (MV) (mm)	3.5 ± 0.7	3.5 ± 0.6	0.61	3.6 ± 0.6	3.5 ± 0.6	0.32
Stent size (MV) ≥ 3.5 mm^a,b^	354 (61%)	104 (65%)	0.39	111 (69%)	104 (65%)	0.41
Stent length (MV) (mm)	26.7 ± 12.6	26.1 ± 13.2	0.57	26.0 ± 13.1	26.1 ± 13.2	0.09
Stent length (MV) ≥ 30 mm	141 (24%)	38 (24%)	0.87	32 (20%)	38 (24%)	0.42
Final balloon size (MV) (mm)	3.5 ± 0.6	3.7 ± 0.6	0.003	3.5 ± 0.6	3.7 ± 0.6	0.004
Maximum balloon size (SV) (mm)	2.5 ± 0.6	–	–	2.4 ± 0.5	–	–

*CTO* chronic total occlusion, *RCA* right coronary artery, *SYNTAX* SYNergy between PCI with TAXus and Cardiac Surgery, *IABP* intra-aortic balloon pumping, *PCPS* percutaneous cardiopulmonary support, *IVUS* intravascular ultrasound, *OCT* optical coherence tomography, *MV* main vessel, *SV* side vessel. Other abbreviations are the same as in Table 1

^a^Potential independent risk-adjusting variables selected for Cox proportional hazards models

^b^Daggers indicate independent model for variables selected for propensity score matching

As a sensitivity analysis, the effects of FKBT strategy relative to non-FKBT strategy were evaluated in the entire study population using the multivariable Cox proportional hazard models, and were expressed as hazard ratios (HRs) with 95% CI. We included 22 clinically relevant factors listed in Tables 1 and 2 as the risk-adjusting variables. Proportional hazard assumptions for the variables were assessed on plots of log (time) versus log (log [survival]) stratified by each variable and were verified as acceptable for all variables. As treatment strategies and other related factors changed over time, 3 periods were defined based on the dominant stent types; bare-metal stent period: 2004–2006; first-generation DES (G1-DES) period: 2007–2009; and second-generation DES (G2-DES) period: 2010–2012. The period was used as a stratification variable.

Two physicians (K. Nishida and M. Toyofuku) and a statistician (T. Morimoto) conducted all the statistical analyses using SPSS version 24 (SPSS, Chicago, IL), JMP version 10.0 (SAS Institute, Cary, NC) and SAS version 9.2 (SAS Institute). All reported *P* values are 2-sided, and *P* values < 0.05 were considered statistically significant.

## Results

### Baseline characteristics

Baseline characteristics were mostly similar between the FKBT and non-FKBT groups in the entire study population, except for the higher prevalence of hemodialysis and peripheral vascular disease in the non-FKBT group. The prevalence of FKBT was significantly different across centers (Table 1). In terms of lesion and procedural characteristics, the prevalence of true bifurcation lesions and mean SYNTAX scores did not differ significantly between the 2 groups (Table 2). Femoral artery was the dominant access site, and G1-DES was implanted in approximately two-thirds of cases without any significant differences between the 2 groups. Patients in the non-FKBT group had significantly higher prevalence of intravascular ultrasound (IVUS) use, as well as lower prevalence of proximal optimization technique, greater number of stents, and larger final balloon size.

In the propensity-matched population of 160 pairs, baseline characteristics were well balanced except for the prevalence of hypertension, peripheral vascular disease, optical coherence tomography (OCT) use, proximal optimization technique, and final balloon size (Tables 1, 2).

### Clinical outcomes: FKBT versus non-FKBT

**Fig. 2:**
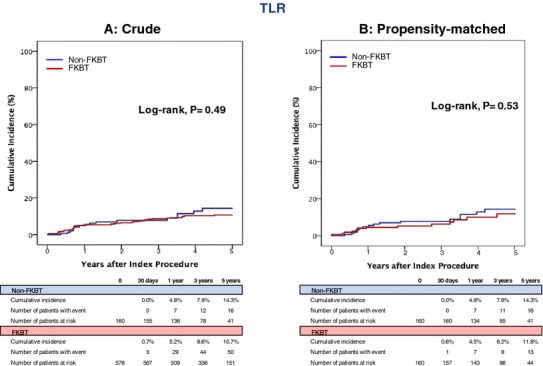
Kaplan–Meier curves for TLR in the entire study population and in the propensity-matched population: FKBT versus Non-FKBT. *FKBT* final kissing balloon technique, *TLR* target lesion revascularization

**Fig. 3:**
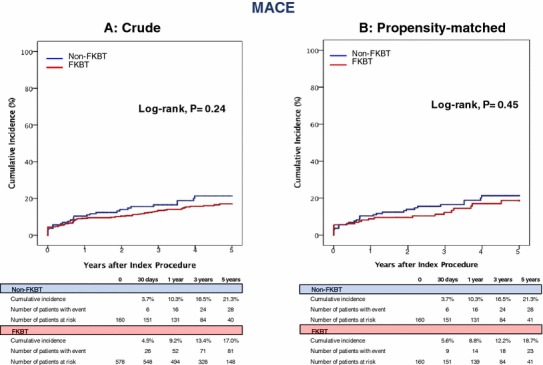
Kaplan–Meier curves for MACE in the entire study population and in the propensity-matched population: FKBT versus Non-FKBT. *FKBT* final kissing balloon technique, *MACE* major adverse cardiac events

Median duration of follow-up after the procedure was 3.8 (interquartile range: 2.2–5.3) years. Overall, 83.4% of patients in this study underwent follow-up coronary artery angiography regardless of the presence of symptoms. In the entire study population, the cumulative 5-year incidence of the primary outcome measure (TLR) was not significantly different between the FKBT and non-FKBT groups (10.7 versus 14.3%, *P* = 0.49) (Table 3, and Fig. 2). Regarding the TLR location, there were no significant differences in the cumulative incidences of TLR for LMCA-only, for the main branch, and for the side branch between the 2 groups (2.2 versus 1.3%, *P* = 0.93, 11.8 versus 9.1%, *P* = 0.71, and 8.2 versus 7.6%, *P* = 0.82, respectively) (Table 3). In the propensity-matched population, the cumulative 5-year incidence of TLR was also not significantly different between the FKBT and non-FKBT groups (11.8 versus 14.3%, *P* = 0.53) (Table 3, and Fig. 2). In the sensitivity analysis by the multivariable Cox proportional hazard model, the effect of FKBT relative to non-FKBT for TLR remained insignificant (adjusted HR 0.89, 95% CI 0.47–1.69, *P* = 0.72) (Table 4). Cumulative 5-year incidences of the secondary outcome measures including MACE were also not significantly differences between the FKBT and non-FKBT groups both in the entire study population and in the propensity-matched population (Table 3 and Fig. 3). The effects of FKBT relative to non-FKBT for the secondary outcome measures were also not significant (Table 4).

**Table 3:** Five-year clinical outcomes in the entire study population and in the propensity-matched population: non-FKBT and FKBT

	Entire study population			Propensity-matched population		
	Patients with at least 1 event (cumulative 5-year incidence, %)			Patients with at least 1 event (cumulative 5-year incidence, %)		
	FKBT (*n* = 578)	Non-FKBT (*n* = 160)	*P* value	FKBT (*n* = 160)	Non-FKBT (*n* = 160)	*P* value
TLR	59 (10.7)	17 (14.3)	0.49	17 (11.8)	17 (14.3)	0.53
TLR-LMCA only	7 (1.3)	2 (2.2)	0.93	1 (0.6)	2 (2.2)	0.56
TLR-main branch	54 (9.1)	15 (11.8)	0.71	14 (10.4)	15 (12.5)	0.5
TLR-side branch	41 (7.6)	11 (8.2)	0.82	10 (6.7)	11 (8.2)	0.59
All-cause death	98 (19.9)	36 (23.1)	0.23	29 (21.2)	36 (23.1)	0.6
Cardiac death	30 (6.3)	18 (9.1)	0.14	11 (8.1)	18 (9.1)	0.68
Sudden death	9 (1.9)	5 (2.0)	0.61	2 (1.5)	5 (2.0)	0.64
Myocardial infarction	12 (2.6)	8 (6.4)	0.06	6 (4.1)	8 (6.6)	0.57
Definite or probable stent thrombosis	2 (0.3)	1 (0.6)	0.62	2 (1.3)	1 (0.6)	0.57
Stroke	25 (5.0)	6 (4.5)	0.87	7 (4.2)	6 (4.5)	0.75
Ischemic	21 (4.2)	3 (2.2)	0.51	5 (2.8)	3 (2.2)	0.99
Hemorrhagic	4 (0.8)	3 (2.2)	0.16	2 (1.4)	3 (2.2)	0.64
Any coronary revascularization	160 (29.9)	42 (34.1)	0.75	39 (26.4)	42 (34.1)	0.24
MACE	92 (17.0)	34 (21.3)	0.24	27 (18.7)	34 (21.3)	0.45

The number of patients with at least 1 event was counted through the entire follow-up period, while the cumulative incidence was truncated at 5 years

*P* values estimated by the log-rank test

*MACE* major adverse cardiac events, *TLR* target lesion revascularization

**Table 4:** Effects of FKBT relative to non-FKBT for clinical outcomes in the crude population

	Unadjusted HR (95% CI)	*P* value	Adjusted HR (95% CI)	*P* value
TLR	0.82 (0.47–1.4)	0.49	0.89 (0.47–1.69)	0.72
All-cause death	0.78 (0.51–1.18)	0.24	0.69 (0.43–1.12)	0.14
Cardiac death	0.6 (0.31–1.19)	0.15	0.41 (0.18–0.94)	0.03
Sudden death	0.71 (0.19–2.67)	0.61	0.31 (0.06–1.56)	0.15
Myocardial infarction	0.42 (0.16–1.01)	0.07	0.55 (0.17–1.79)	0.32
Definite or probable stent thrombosis	N/A	N/A	N/A	N/A
Stroke	0.93 (0.37–2.3)	0.87	0.89 (0.32–2.46)	0.82
Ischemic	1.5 (0.44–5.12)	0.52	1.15 (0.29–4.5)	0.84
Hemorrhagic	0.36(0.08–1.59)	0.18	0.71 (0.12–4.29)	0.71
Any coronary revascularization	0.95 (0.67–1.34)	0.75	0.81 (0.55–1.2)	0.3
MACE	0.78 (0.51–1.19)	0.25	0.64 (0.39–1.06)	0.08

Effect of FKBT relative to non-FKBT is expressed as a hazard ratio with the 95% confidence interval by Cox proportional hazard models

*CI* confidence interval, *HR* hazard ratio, *N/A* not assessed. Other abbreviations are the same as in Table 3

## Discussion

The main findings of the present study from a large multicenter registry in Japan were the followings; (1) FKBT after a 1-stent strategy for LMCA crossover stenting did not affect TLR and other clinical outcomes during 5-year follow-up; (2) FKBT also did not affect the location of TLR (Fig. 3).

FKBT has been widely performed at any bifurcation on the expectations of optimizing stent apposition at the main branch, ameliorating the side branch ostial narrowing caused by carina shift, reducing strut protrusion into the side branch ostium, and maintaining access to the side branch [16]. In contrast, FKBT might be associated with injury of the side branch ostium potentially leading to restenosis, stent deformation related to over-dilation of the stent proximal to the side branch, and strut mal-apposition due to inadequate rewiring position [17, 18]. FKBT is generally advocated after a two-stent strategy for any bifurcation [19, 20]. However, the clinical benefits of routine FKBT after a 1-stent strategy appear uncertain at any bifurcation lesion according to the several previous studies [8–11]. The role of FKBT at LMCA bifurcation might be markedly different from that at any other bifurcation, because LMCA bifurcation involves LCX, which is often a large vessel and supplies a large myocardial territory. FKBT would be justified in cases of hemodynamically significant ostial LCX stenosis or flow impairment of LCX after crossover stenting. However, it remains uncertain whether FKBT should be performed in cases of preserved LCX flow or without significant stenosis at ostial LCX ostium after LMCA crossover stenting.

Crossover stenting for the LMCA as a 1-stent strategy is forced to jail the LCX ostium by stent struts. There were concerns on the possibility that formation of neointima and thrombus at the jailed strut with compromise of LCX flow may increase the risk of ST or TLR [21]. A OCT study noted that FKBT may reduce the frequency of uncovered struts and subclinical thrombus at the side branch orifice, although FKBT did not decrease MACE and TLR in this small cohort [22]. The COBIS II registry, which included LMCA lesions in 26% of cases, demonstrated that a 1-stent technique with FKBT for any bifurcation lesions was associated with favorable long-term clinical outcomes, whereas a 1-stent strategy with FKBT for LMCA was not associated with better MACE outcomes compared with a 1-stent strategy without FKBT [11]. Furthermore, two recent single center registries found that midterm clinical outcomes were not significantly different regardless of FKBT after crossover stenting of the LMCA [12, 13]. As in the previous reports, the present multicenter study with longer follow-up, with a higher frequency of FKBT, and with TLR as the primary outcome measure did not demonstrate superiority of FKBT strategy over non-FKBT strategy.

Interestingly, the frequency of FKBT after crossover stenting for the LMCA in the present study was higher (78%) than those reported in previous studies (32–35%) [11, 12]. Reflecting the operator preferences, there is a wide variation across countries and institutions, regarding the performance of FKBT after crossover stenting for LMCA. The reason for the high frequency of FKBT in our registry might be that FKBT has been mainly performed not only for decreased coronary blood flow, but also for opening the jailed strut with FKBT to maintain access to the LCX for future PCI. The rationale for this “prophylactic” FKBT would be a concern about stent deformation and restenosis caused by balloon dilatation for a stable jailed strut of the LCX ostium and for stable LMCA in cases that need TVR for LCX in the future.

Despite concerns of increased TLR rate for the proximal LMCA regarding polymer damage, strut deformation, and asymmetry of the proximal stent by the hugging balloon, which potentially decrease tissue concentrations of the eluted drug, TLR rate for the proximal LMCA did not differ significantly regardless of FKBT in the present study [23]. The frequency of TLR for LCX involving the ostium was similar with or without FKBT in the present study, despite concerns about balloon injury or incomplete stent apposition with FKBT. Therefore, FKBT would not be harmful in terms of midterm outcomes in this study. One of the reasons for these neutral results might be that FKBT was not harmful despite its potential risks, because the frequency of IVUS use was higher than in previous reports [9–12]. Guidance with IVUS or OCT is useful particularly for the LMCA for confirming side branch distal rewiring before FKBT and determination of stent and balloon sizes [18, 24]. However, FKBT often needs a higher volume of contrast, longer procedure time, and more devices than no FKBT [9, 12]. Based on our present results, FKBT may not be mandatory in cases without flow limitation after stenting. Further and longer term validation study would be required for this issue.

### Limitations

The present study has several important limitations. First, this registry was conducted as a nonrandomized and retrospective observational study. Therefore, we performed propensity-matched analysis to adjust for the potential confounders. Nevertheless, we could not deny the presence of unmeasured confounders and selection bias. Second, we did not assess the anatomic assessments such as the extent of myocardial territory and of previous myocardial infarction or diameter of LCX, and the degree of stenosis at the LCX ostium after crossover stenting, which might be closely related to the decision whether to perform FKBT. Particularly, significance of LCX territory or viability may affect the clinical outcome. Additionally, patients treated with 2-stent strategy for bailout subsequent to FKBT was excluded in this study cohort, who might have been benefitted from LCX intervention. Third, physiological assessment such as fractional flow reserve was not adequately analyzed, although angiography often overestimate the stenosis of the LCX ostium compared with functional assessment [25]. Fourth, the frequency of follow-up coronary artery angiography was high compared to previous reports, which might have increased the incidence of angiography-driven TLR. Fifth, the sample size was not large enough to evaluate those clinical outcomes such as ST, MI, and cardiac death. Indeed, the risk for MI and cardiac death favored FKBT, although we could not draw definitive conclusions. Finally, G1-DES were used in a large proportion of patients. Use of newer generation DES has been shown to improve clinical outcomes after LMCA stenting [4, 5].

## Conclusion

FKBT after a 1-stent strategy for LMCA crossover stenting did not affect TLR and other clinical outcomes during 5-year follow-up.

## Electronic supplementary material

Below is the link to the electronic supplementary material.

Supplementary material 1 (DOCX 26 kb)
