# Supplementary material for: Prognostic impact of final kissing balloon technique after crossover stenting for the left main coronary artery: from the AOI-LMCA registry
Source: Cardiovasc Interv Ther. 2018 Apr 24;34(3):197–206. doi: 10.1007/s12928-018-0522-0 (PMC6561992; doi:10.1007/s12928-018-0522-0)
Supplement: Supplementary file 1 — Supplementary material 1 (DOCX 26 kb) [file 12928_2018_522_MOESM1_ESM.docx]

**Supplemental Table.** Coefficients of the independent variables in the logistic regres

sion function

| Variable | Coefficient |
| --- | --- |
| Intercept | -0.4810 |
| Age ≥80 | -0.6592 |
| Diabetes mellitus | -0.5119 |
| Hemodialysis | 0.0089 |
| eGFR <60 mL/min/1.73 m^2^ and non-hemodialysis | 0.1520 |
| Previous heart failure | 0.4011 |
| Peripheral vascular disease | 0.6107 |
| UAP/NSTEMI | -0.0055 |
| Time period: Wave 2; 2007-2009 (G1-DES period) | 0.2093 |
| Time period: Wave 3; 2010-2012 (G2-DES period) | 0.5368 |
| Institute 1 | -0.2371 |
| Institute 2 | 0.3665 |
| Institute 3 | -2.4889 |
| Institute 4 | -0.1397 |
| Institute 5 | -0.6849 |
| CTO in the RCA | 0.6286 |
| True bifurcation | -0.0823 |
| Multi-vessel (left main + ≥2 vessels) | -0.3869 |
| Calcified lesion | 0.0293 |
| Stent type: G2-DES | -0.1355 |
| Use of intracoronary imaging modalities: IVUS | 0.3782 |
| Stent size (MV) ≥ 3.5 mm | -0.2513 |
